# Characterization of the Ubiquitin‐Modified Proteome of Recombinant Chinese Hamster Ovary Cells in Response to Endoplasmic Reticulum Stress

**DOI:** 10.1002/biot.202400413

**Published:** 2024-12-02

**Authors:** Karuppuchamy Selvaprakash, Christiana‐Kondylo Sideri, Michael Henry, Esen Efeoglu, David Ryan, Paula Meleady

**Affiliations:** ^1^ Life Sciences Institute Dublin City University Dublin Ireland; ^2^ School of Biotechnology Dublin City University Dublin Ireland; ^3^ SSPC the SFI Research Centre for Pharmaceuticals Dublin City University Dublin Ireland

**Keywords:** Chinese hamster ovary (CHO) cells, endoplasmic reticulum stress, K‐ε‐GG peptides, LC‐MS/MS analysis, proteasome inhibition, ubiquitination

## Abstract

Chinese hamster ovary (CHO) cells remain the most widely used host cell line for biotherapeutics production. Despite their widespread use, understanding endoplasmic reticulum (ER) stress conditions in recombinant protein production remains limited, often creating bottlenecks preventing improved production titers and product quality. Ubiquitination not only targets substrates (e.g., misfolded proteins) for proteasome degradation but also has important regulatory control functions including cell cycle regulation, translation, apoptosis, autophagy, etc. and hence is likely to be central to understanding and controlling the productivity of recombinant biotherapeutics. This study aimed to uncover differentially expressed ubiquitinated proteins following artificial induction of ER‐stress in recombinant CHO cells. CHO cells were treated with the stress inducer tunicamycin and the proteasome inhibitor MG132, followed by LC‐MS/MS proteomic analysis. We identified >4000 ubiquitinated peptides from CHO‐DP12 cells under ER stress conditions and proteasome inhibition. Moreover, data analysis showed altered abundance levels of >900 ubiquitinated proteins under the combination of ER stress and proteasome inhibition compared to untreated controls. Gene Ontology (GO) analysis of these ubiquitinated proteins resulted in a significant enrichment of key pathways involving the proteasome, protein processing in the ER, *N*‐glycan biosynthesis, and ubiquitin‐mediated proteolysis. ER stress response proteins such as GRP78, HSP90B1, ATF6, HERPUD1, and PDIA4 were found to be highly ubiquitinated and exhibited a significant increase in abundance following induction of ER‐stress conditions. This study broadens our comprehension of the roles played by protein ubiquitination in CHO cell stress responses, potentially revealing targets for tailored cell line engineering aimed at enhancing stress tolerance and production efficiency.

## Graphical Abstract and Lay Summary

Ubiquitination is an essential cellular process in which proteins are tagged with ubiquitin, marking them for proteasomal degradation. Beyond this role, ubiquitination plays key functions in ER stress, cell signalling, autophagy, and cell cycle regulation. To gain deep insights into ER stress mechanisms and changes to the ubiquitinated proteome of recombinant Chinese hamster ovary (CHO) cells, CHO‐DP12 cells were treated with the ER‐stress inducer tunicamycin, the proteasome inhibitor MG132, and a combination of both. Ubiquitinated proteomic analysis using quantitative label‐free LC‐MS/MS identified many differentially abundant ubiquitinated proteins following exposure to these compounds. The study has the potential for manipulation of pathways such as the UPR and ERAD to reduce misfolded proteins, improve folding efficiency, and optimize cellular health during bioproduction processes.

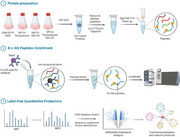

## 1 Introduction

Biotherapeutics, including monoclonal antibodies (mAbs), are experiencing remarkable market growth in the biopharmaceutical industry [1]. Chinese hamster ovary (CHO) cells remain the preferred mammalian host cell line for the production of biotherapeutics [1, 2, 3]. This preference is attributed to their ability to grow in suspension culture at high cell densities, carry out precise protein folding and human‐like posttranslational modifications (PTMs), and adapt to diverse cell growth conditions [3]. Protein therapeutics, particularly mAbs, continue to lead the sales and the number of new approved products from CHO cells [1]. Although CHO cell platforms have been extensively employed to produce biotherapeutics, intrinsic limitations remain in the synthesis and secretion of many complex and difficult‐to‐express biotherapeutics [4, 5]. The limitations include low productivity, low resistance to culture‐related stresses, and high production costs [6]. These challenges arise due to various factors affecting intracellular protein processing, such as the cellular secretory capacity, protein folding, and protein aggregation or degradation [6]. To overcome these challenges, targeted engineering strategies are actively pursued to further optimize current CHO cell systems [7]. Consequently, an increased understanding of the cellular physiology of CHO cells is of critical importance to enhance the production capabilities and efficiency of the host cell line.

Studies have highlighted the posttranslational bottlenecks in the biosynthetic pathway of biotherapeutics in CHO cells [8]. These studies suggest that targeting the protein secretion pathway machinery in CHO cells can lead to increased expression levels of mAbs [9, 10, 11, 12]. The endoplasmic reticulum (ER), a central organelle in the protein secretion pathway, plays a crucial role in regulating various processes, including protein synthesis, translocation, and early PTMs [13]. Excessive protein production, nutrient deprivation, and other cell culture media conditions in CHO cells can lead to protein unfolding and misfolding, which in turn triggers ER stress [14]. When ER stress occurs, cells initiate the unfolded protein response (UPR) pathway to restore protein homeostasis and the folding capacity of the ER [14]. When exposed to low levels of stress, the cells can adapt and manage the increased ER load. However, under conditions of high ER stress, when cellular homeostasis cannot be restored, the UPR may trigger apoptosis [15]. Although CHO cells have strongly established their position in the industrial setting to produce recombinant proteins, gaining deeper insights into the relationship between ER stress and key pathways, such as the UPR, endoplasmic reticulum‐associated degradation (ERAD), and the ubiquitin‐proteasome system (UPS), could further improve production efficiency.

Several proteomic studies have been conducted in CHO cells by our group [16, 17, 18] and others [19, 20, 21, 22], aiming to understand phenotypes related to growth and productivity in different culture conditions. Nevertheless, the majority of ‘omic‐based studies in CHO cells often neglect to consider cellular PTMs, despite their crucial role in influencing the functionality of a protein. There have been a few studies starting to emerge on the analysis of the phosphoproteome in CHO cells in various bioprocess‐related conditions such as growth, productivity, and temperature shift [9, 11, 17, 23, 24, 25]. Moreover, the glycoproteome of CHO cells, characterizing N‐ and O‐linked glycosylation of cellular proteins, has been studied to investigate the role of glycosylation in various cellular functions, including cell adhesion, signaling, and immune responses [26, 27, 28, 29]. Ubiquitination is one of the most important PTMs and involves the covalent attachment of ubiquitin to a lysine residue of a target protein which is essential to numerous aspects of cellular physiology [30]. The primary role of ubiquitination is to regulate protein stability by marking damaged or surplus proteins for degradation via the proteasome or lysosome, ensuring their removal [30]. Additionally, ubiquitination controls protein activity, localization, and interactions, impacting vital cellular processes such as signaling, DNA repair, autophagy, cell death, and receptor trafficking [30, 31]. Ubiquitination also plays a central role in maintaining cellular homeostasis and orchestrating diverse cellular responses. Despite the evident significance of ubiquitination in such protein signaling pathways [32, 33, 34], the ubiquitinated proteome in CHO cell lines remains understudied. There is also a growing body of evidence indicating that the UPS pathway plays a pivotal role in regulating the growth, productivity, and product quality of recombinant CHO cells. Therefore, enhancing our understanding of the fundamental role of protein ubiquitination in proteasomal degradation, cell signaling, autophagy, and cell cycle regulation within CHO cells could open up new avenues for engineering CHO cell lines to exhibit improved bioprocess phenotypes. Moreover, a global differential abundance study of PTMs, particularly ubiquitination, holds great significance in gaining deeper insights into the proteome of CHO cells in bioprocess conditions to potentially improve the efficacy of biotherapeutic production.

In this study, we conducted a ubiquitinated proteomic analysis of anti‐IL8‐IgG1‐producing CHO‐DP12 cells treated with tunicamycin (TM) to artificially induce ER stress. (Note that this study focuses on changes in the abundance of ubiquitinated proteins, rather than on specific alterations in the ubiquitin load on individual proteins, as a result of treatment with TM and a combination of TM and MG132). TM is a UPR inducer that disrupts the first step of N‐linked protein glycosylation in the ER by inhibiting GlcNAc‐1‐P‐transferase [33, 35]. This disruption leads to extensive protein misfolding and ER stress. To enhance and preserve protein ubiquitination in CHO‐DP12 cells, we used MG132 as a proteasomal inhibitor. MG132 inhibits the 26S‐proteasome activity, thereby preventing the degradation of ubiquitinated proteins [36, 37]. This inhibition enables us to investigate protein turnover, degradation pathways, and the role of the proteasome in various cellular processes in more detail. Additionally, to gain deep insights into ER stress and changes to the ubiquitinated proteome, we treated CHO‐DP12 cells with TM or MG132 on their own and also with a combined treatment of TM and MG132. Ubiquitinated peptide enrichment from protein digests in combination with quantitative label‐free LC‐MS/MS proteomic analysis of the whole cell proteome was used to compare the differential abundance of ubiquitinated proteins following exposure to TM, MG132, and combinations of both compounds. Furthermore, we conducted Gene Ontology (GO) analysis on the resultant proteins to identify enriched biological processes and KEGG pathways in CHO‐DP12 cells.

## 2 Materials and Methods

### 2.1 Reagents and Materials

BalanCD CHO Growth A media was obtained from FUJIFILM (Fujifilm Europe B.V, NL). l‐Glutamine, dithiothreitol (DTT), iodoacetamide (IAA), methotrexate (MTX), bovine serum albumin (BSA), and TM were purchased from Sigma–Aldrich (Merck, IE). ProteaseMAX surfactant was obtained from Promega (MyBio, IE). Acetonitrile (ACN), dimethylsulfoxide (DMSO), trifluoroacetic acid (TFA), Pierce660‐nm Protein Assay Kit, sequencing grade trypsin, trypan blue stain (0.4%), and Lys‐C were obtained from Thermo Fisher Scientific (Biosciences, IE). Sep‐Pak C18 Cartridges were purchased from Waters (Waters Company, UK). PTMScan HS Ubiquitin/SUMO Remnant Motif (K‐ε‐GG) kit was acquired from Cell Signaling Technologies (Brennan & Co., IE).

### 2.2 Cell Culture

CHO‐DP12 cell line (ATCC CRL‐12445 clone #1934), which produces an anti‐IL8‐IgG1, was used for all experiments. These cells are DHFR‐deficient and require MTX to maintain selection and for gene amplification to enhance the production of the IL‐8 mAb. Cells were grown in BalanCD CHO Growth A media supplemented with 4 mM l‐glutamine at 37°C under 5% CO_2_ and 80% humidity, in a Climo‐Shaker ISF1‐X orbital shaker (Kühner, CH). Cell lines were routinely tested for mycoplasma contamination and were found to be negative. Every 2–3 weeks, cells were pulsed with 400 nM MTX.

### 2.3 Tunicamycin and MG132 Treatment of CHO‐DP12 Cells

Cells were seeded at 8 × 10^5^ cells/mL in a 30 mL final volume using 250 mL flat‐bottomed Erlenmeyer flasks. The total cell count and viability were determined using an Invitrogen Countess cell counter using trypan blue reagent (Thermo Fisher Scientific, UK). The seeded cells were incubated in the orbital shaker for 24 h, followed by the addition of 10 µg/mL TM or 0.1 µM MG132. The concentration of TM and MG132 was chosen based on achieving a sufficient induction of ER stress and minimizing cell death over a 24‐h exposure period [38, 39]. The equivalent volume of DMSO was added to the control cell culture batch. The amount of DMSO added was nontoxic to the cells [39]. Cells were further incubated with TM, MG132, and TM + MG132 for 24 h and pelleted. For pelleting, cells were washed three times with ice‐cold PBS to remove any residue from the cell culture media components and stored at −80°C before MS sample preparation. Three independent cell pellets were obtained from three independent subcultures for each set of treatments (i.e., CHO‐DP12 + TM, CHO‐DP12 + MG132, and CHO‐DP12 + TM + MG132*)*.

### 2.4 Preparation of Cell Lysates and Protein Digestion for LC‐MS Analysis

Approximately 1 × 10^8^ cells were lysed in 8 M urea, 50 mM Tris, and 100 mM NaCl (pH 8.5) buffer containing 0.04% ProteaseMAX surfactant detergent. Cell lysis was performed on ice using a handheld ultrasonicator with short sonication pulses (×3 cycles) per sample. After obtaining a homogenous mixture, the cell debris was removed by centrifugation at 14,000 × *g* for 10 min at 4°C. The protein‐containing supernatant was transferred to a new microcentrifuge tube. The protein quantification assay was performed using the Pierce 660 nm assay according to the manufacturer's instructions, and BSA was used as the protein standard at various concentrations.

Five milligrams of protein was used from each sample for proteomic and ubiquitinated proteomic analysis. Disulfide bonds were reduced by incubating the protein samples with 5 mM DTT for 30 min at 56°C, followed by alkylation with 10 mM IAA at room temperature for 30 min in the dark. Alkylated samples were further diluted five‐fold in 50 mM ammonium bicarbonate (NH_4_HCO_3_) (pH ∼8.5) to reduce the urea concentration from 8 to 1.6 M for peptide digestion. Proteins were initially digested with Lys‐C (1:200 Lys‐C:protein ratio, w/w) for 4 h at 37°C followed by trypsin (1:100 trypsin:protein ratio, w/w) overnight at 37°C. After overnight digestion, TFA was added to a final concentration of 0.5% to quench the remaining activity of trypsin. Digested samples containing the peptide mixture were desalted using a Sep‐Pak C18 cartridge. Briefly, the C18 cartridge was first conditioned by adding 5 mL of ACN followed by 3 mL of 50% ACN/0.1% TFA. The cartridge was then equilibrated with 5 mL of 0.1% TFA, and the samples were loaded with 0.5% TFA. Loaded samples were desalted with 5 mL of 0.1% TFA. Desalted peptides were eluted with 5 mL of 50% ACN/0.1% TFA. The eluted samples were lyophilized separately into two portions, that is, 0.5 mL (for peptide analysis) and 4.5 mL (for K‐ε‐GG peptide enrichment) and stored at −80°C until use.

### 2.5 Ubiquitin diGLY Peptide Enrichment

The ubiquitin diGLY peptide enrichment was carried out using ubiquitin remnant motif (K‐ε‐GG) antibodies coupled to magnetic beads according to the manufacturer's instructions. Lyophilized peptides were dissolved in 1.5 mL of PTMScan HS immunoaffinity purification (IAP) bind buffer (1X). The pH of the peptide solution after dissolution was checked to ensure the pH was lower than 7. To ensure a clear solution, the peptide mixture was centrifuged for 1 min at 10,000 × *g* at 4°C and subsequently cooled on ice. Next, the vial of the antibody‐bead slurry was centrifuged at 2000 × *g* for 1 min to bring down any beads clinging to the sides and cap of the vial. To prepare the antibody beads, 20 µL of the bead slurry was taken and washed with 1 mL of ice‐cold PBS (1X). The bead slurry was centrifuged again at 2000 × *g* and the supernatant was removed. The soluble peptide solution (1.5 mL) was incubated on an end‐over‐end rotator for 2 h at 4°C. After the incubation, the tube was centrifuged at 2000 × *g* for 1 min followed by magnetic separation for 3 min. The beads were washed (×3) with PTMScan HS IAP wash buffer (1X) and ice‐cold LC‐MS water (×3). Peptides bound to the beads were eluted with 100 µL of 0.2% TFA by incubating at room temperature for 10 min. The elution step was repeated with an additional 100 µL of 0.2% TFA. Finally, eluted peptides were desalted using a C18 stage tip and dried using a vacuum evaporator.

### 2.6 Nano LC‐MS/MS

Mass spectra were acquired on a hybrid linear ion trap/Orbitrap Fusion Tribrid mass spectrometer (Thermo Fisher Scientific, UK) coupled to a Dionex Ultimate 3000 RSLCnano system (Thermo Fisher Scientific, UK). PepMap100 (C18, 300 µm × 5 mm) and Acclaim PepMap 100 (5 µm × 50 cm, 3 µm bead diameter) columns were used as trapping and analytical columns, respectively. K‐ε‐GG peptide and non‐K‐ε‐GG peptide enriched samples were resolubilized in 20 µL of 0.1% formic acid and 2% ACN in LC‐MS grade water. Peptide concentration in each sample was determined using a NanoDrop One (Thermo Scientific) and the preprogrammed scopes application measuring at 205 nm. One microgram of peptides from each sample were loaded onto the trapping column at a flow rate of 25 µL/min with 2% (v/v) ACN and 0.1% (v/v) TFA for 3 min. Samples were resolved in the analytical column using a binary gradient of formic acid solution (0.1% [v/v] formic acid in LC‐MS grade water) and ACN/formic acid solution (80% (v/v) ACN, 0.08% (v/v) formic acid in LC‐MS grade water). A flow rate of 280 nL/min was used to elute the peptides. For all experiments, the MS instrument was operated in data‐dependent acquisition (DDA) mode using a full scan range of 380–1500 *m*/*z*, and each sample was run for 140 min. MS1 spectra were collected at a resolution of 120,000 with an automated gain control (AGC) target 2 × 10^5^ with a maximum injection time of 50 ms. The number of selected precursor ions for fragmentation was determined using a top‐speed acquisition algorithm, and ions were isolated in the quadrupole using an isolation width of 1.6 Da. Fragmentation of precursor ions was carried out using high‐energy collision‐induced dissociation, and the resulting MS/MS ions were detected via a linear ion trap. Dynamic exclusion was applied to analyze peptides after 60 s, and peptides with a charge state between 2+ and 7+ were analyzed. Peptides were fragmented via higher energy collision‐induced dissociation with a collision energy of 28, an AGC value of 1 × 10^5^, and a maximum injection time of 90 ms. Using Sequest search engine and Percolator in Thermo Scientific Proteome Discoverer 2.2 software against a Cricetulus griseus database (downloaded from UniProt in January 2023), the following search parameters were used for the protein identifications: (1) peptide and fragment mass tolerance were set to 10 ppm and 0.6 Da respectively, (2) up to three missed cleavages were allowed, (3) carbamidomethylation of cysteine (125.0476 Da) was set as a fixed modification, (4) oxidation of methionine (15.99491 Da), and (5) N‐terminal acetylation (125.0476 Da) were set as variable modifications. For the identification of ubiquitinated enriched samples, lysine with a K‐ε‐GG remnant (114.0429 Da) was additionally set as a variable modification to the same search parameters.

### 2.7 Differential Ubiquitinated Proteomic Analysis

Raw LC‐MS/MS data files were processed using Progenesis QI for Proteomics software version 2.0 (Nonlinear Dynamics, Waters, Newcastle upon Tyne, UK). Briefly, after aligning peptide LC retention times from all MS data files to an assigned reference run, the samples from the two experimental groups (Group 1: control; Group 2: cells treated with TM or MG132 or TM + MG132) were set up within the Progenesis software for analysis of altered abundance levels of ubiquitinated peptides and proteins. Label‐free quantitation was carried out for each experimental group after peak detection and normalization. To identify the list of ubiquitinated proteins with altered abundance levels, peptide features were filtered using the following parameters: (1) peptide features with ANOVA ≤0.05 and (2) minimum fold‐change of relative peptide abundance between the two experimental groups ≥ ± 1.5‐fold. The filtered MS data was exported as a; .mgf file for protein identification using Proteome Discoverer software version 2.2 (Thermo Fisher Scientific, UK). A database search was performed using Sequest HT against a Cricetulus griseus database (downloaded from UniProt in January 2023). The identification results were exported as a pep.xml file and imported back into Progenesis QI for Proteomics. For analysis of altered abundance levels of ubiquitinated proteins, only high confident peptide identifications with an FDR ≤ 0.01 were imported into Progenesis QI software. Raw MS data files are available for download from ProteomeXchange Consortium via the PRIDE partner repository with the dataset identifier PXD049241.

### 2.8 Gene Ontology

Ubiquitinated proteins with altered abundance levels identified from Progenesis QI for Proteomics were imported into STRING GO (https://version‐12‐0.string‐db.org /, accessed on July 2023) to determine enriched biological processes, KEGG pathways, molecular functions, as well as protein–protein interactions. Strength and FDR were used to determine the most significant enrichments. The measure “Strength” describes the size of the enrichment effect. It is the ratio of (i) the number of proteins in the network annotated with a specific term and (ii) the number of proteins expected to be annotated with that term in a random network of the same size. *p* values were corrected for multiple testing within each category using the Benjamini–Hochberg procedure. Proteins that are enriched with a *p* value of <0.05 were included.

## 3 Results and Discussion

### 3.1 Induction of ER Stress in Recombinant CHO Cells

**FIGURE 1:**
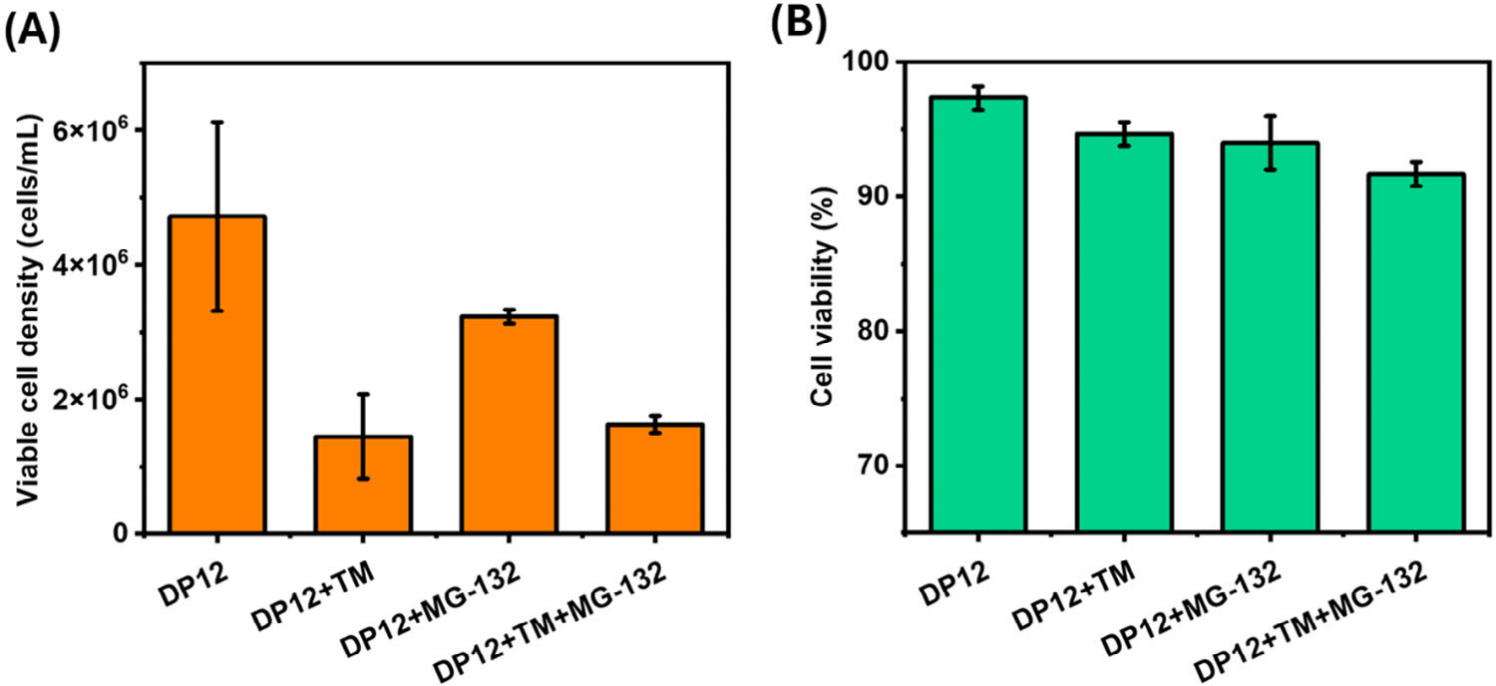
(A) Viable cell density (orange) and (B) viability (green) of CHO‐DP12 cells after treatment with TM, MG‐132, and TM + MG132 combined. CHO, Chinese hamster ovary; TM, tunicamycin.

In order to induce ER stress, CHO‐DP12 cells were initially treated with TM. TM is a canonical UPR inducer that blocks N‐linked glycosylation of proteins leading to an increase in the number of misfolded proteins [36, 37]. Initially, we evaluated the impact on cell growth and culture viability in the presence of TM and MG132. Figure 1 shows the effect of the ER stressor and the proteasome inhibitor as well as their combination on CHO cell growth and viability. The cell viability was not affected significantly upon TM, MG132, and TM + MG132 treatments, whereas cell growth was observed to slow down in the cells that were exposed to TM, MG132, and the combination of both. To confirm the induction of ER stress response in CHO‐DP12 cells by TM, we assessed the expression levels of UPR‐associated stress marker proteins by label‐free proteomic analysis comparing the control cells to the treated cells. This was accomplished through LC‐MS/MS analysis, followed by differential expression analysis using the Progenesis QI for Proteomics software. Increased abundance levels of UPR markers [40] such as GRP78 (4‐fold, ANOVA *p* < 0.05), HSP90B1 (2.4‐fold, ANOVA *p* < 0.05), and PDIA4 (2.6‐fold, ANOVA *p* < 0.05) after TM treatment can be observed in Supporting Information File S2. The abundance levels of each of these proteins were significantly increased in the TM‐treated samples confirming the activation of the UPR in the CHO‐DP12 cell line.

### 3.2 Enrichment of Ubiquitinated Proteins From Recombinant CHO Cells

Modified peptides/proteins often have relatively lower stoichiometry compared to their nonmodified counterparts. When measuring modified peptides or proteins, enrichment steps are necessary to overcome the low abundance of PTMs like ubiquitination. Moreover, it is also essential to preserve and enrich the low abundance PTMs such as ubiquitination in CHO cells for an in‐depth understanding of their role in recombinant protein production. The proteasome inhibitor MG132 inhibits the activity of proteasomes [41], which are essential proteolytic complexes responsible for degrading ubiquitinated proteins in the cells. By blocking the proteasome's function, MG132 reduces the breakdown of ubiquitin‐conjugated proteins, leading to their accumulation within the cell [41, 42]. In order to boost and preserve the protein ubiquitination in CHO‐DP12, the proteasomal inhibitor MG132 was added to the CHO‐DP12 cells. To compare the effect of ER stress and proteasomal inhibition, a combination of TM and MG132 was also added. Protein isolation followed by tryptic digestion was conducted. During the tryptic digestion process, trypsin leaves a specific K‐ε‐GG remnant on the ubiquitinated lysine residue of proteins. This motif generates a distinct mass difference (114.04 Da), enabling unambiguous identification and recognition of ubiquitination sites [43].

**FIGURE 2:**
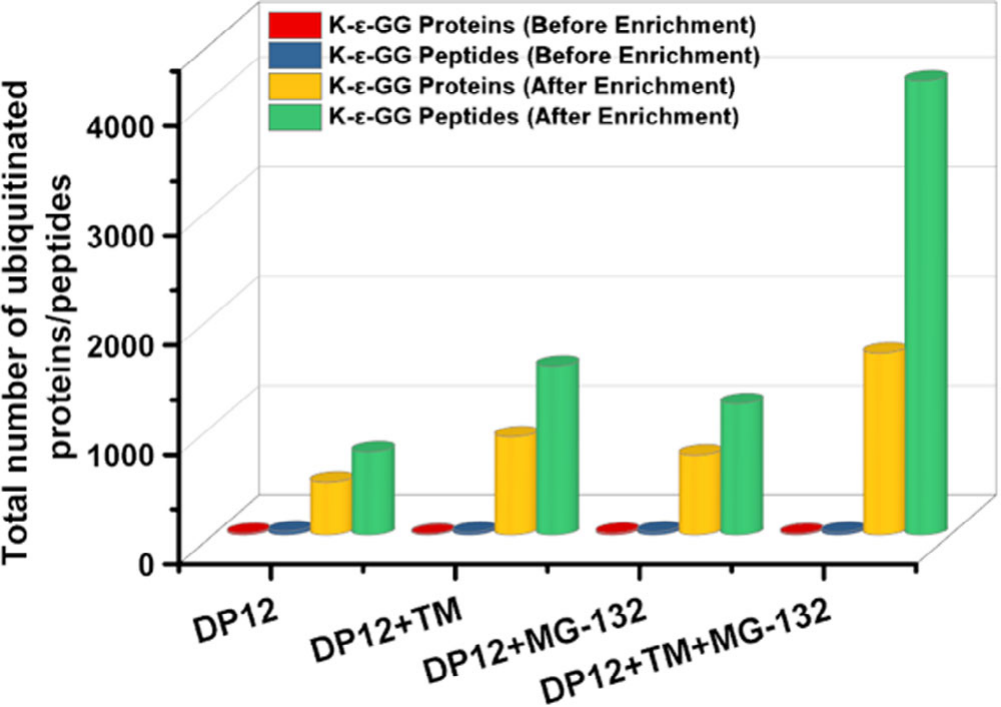
Total number of ubiquitinated peptides and proteins identified from CHO‐DP12 cells after treatment with TM, MG‐132, and TM + MG132 combined. CHO, Chinese hamster ovary; TM, tunicamycin.

To identify proteomic and ubiquitinated proteomic differences before and after ER stress and proteasome inhibition in CHO cells, three independent biological replicates per treatment were prepared for LC‐MS/MS analysis. Figure 2 shows the total number of K‐ε‐GG peptides/proteins detected before and after treatment with TM, MG132, and TM + MG132 using the K‐ε‐GG‐antibodies coupled to beads. Before the differential expression analysis, each LC‐MS file from each sample group was searched against the CHO UniProt database using the SequestTMHT search algorithm in Proteome Discoverer 2.2. The database search of the K‐ε‐GG modification in nonenriched samples from control CHO‐DP12 cells, along with their TM, MG132, and TM + MG132 treated counterparts, resulted in the identification of a very limited number of K‐ε‐GG peptides. Specifically, 34 (untreated), 27 (TM), 30 (MG132), and 27 (TM + MG132) K‐ε‐GG peptides were found, corresponding to 16, 12, 18, and 14 ubiquitinated proteins with unique peptides ≥ 1, respectively (Figure 2). Moreover, in the database search for all ubiquitin‐enriched samples from control, TM, MG132, and TM + MG132 treated counterparts, a more extensive list of K‐ε‐GG peptides was identified. The total numbers of K‐ε‐GG peptides found from this qualitative assessment of the data generated were 760, 1542, 1199, and 4146, corresponding to 482, 897, 731, and 1661 ubiquitinated proteins with unique K‐ε‐GG peptides  ≥ 1, respectively (Figure 2). These findings indicate a significant increase in ubiquitinated peptides/proteins upon enrichment, highlighting the efficiency of the enrichment method in capturing a broader range of ubiquitinated species in the samples. It should be noted that the number of peptides/proteins in TM + MG132 treatment was higher than TM and MG132 alone. During ER stress, specific proteins undergo ubiquitination and are subsequently degraded by the UPS. However, MG132 inhibits this process, leading to protein accumulation [37]. Therefore, the increased protein levels observed during ER stress induced by TM treatment result from enhanced ER stress signaling, activation of the UPR, and the inhibition of ubiquitin‐mediated protein degradation due to proteasomal blockage. In total, approximately 4100 ubiquitinated sites (Supporting Information File S1) were identified from CHO‐DP12 cells following induction of ER stress and proteasomal inhibition.

### 3.3 Differential Ubiquitinated Proteomic Analysis of Recombinant CHO Cells

From various CHO cell line engineering studies, there is a growing body of evidence indicating that the UPS pathway plays a pivotal role in regulating the growth, productivity, and product quality of recombinant CHO cells. For example, it has been shown that the ubiquitin ligase inhibitor thalidomide enhanced the expression of biologically active fusion protein HSA‐HGF in CHO cells [44]. Recent investigations have also revealed altered levels of ubiquitin‐proteasome‐related proteins as direct targets of microRNAs (miRNAs), which have been shown to exert phenotypic effects on cell growth [45] and productivity [46]. In particular, the miR‐30 family targets the ubiquitin E3 ligase Skp2, resulting in an enhancement of productivity in CHO cells [43]. Recombinant protein productivity is sensitive to cellular stress, often leading to protein misfolding and subsequent dysfunction. This degradation of short‐lived and dysfunctional proteins in CHO cells is primarily managed by the UPS pathway. This pathway plays a crucial role in maintaining protein quality and cellular homeostasis [47].

**FIGURE 3:**
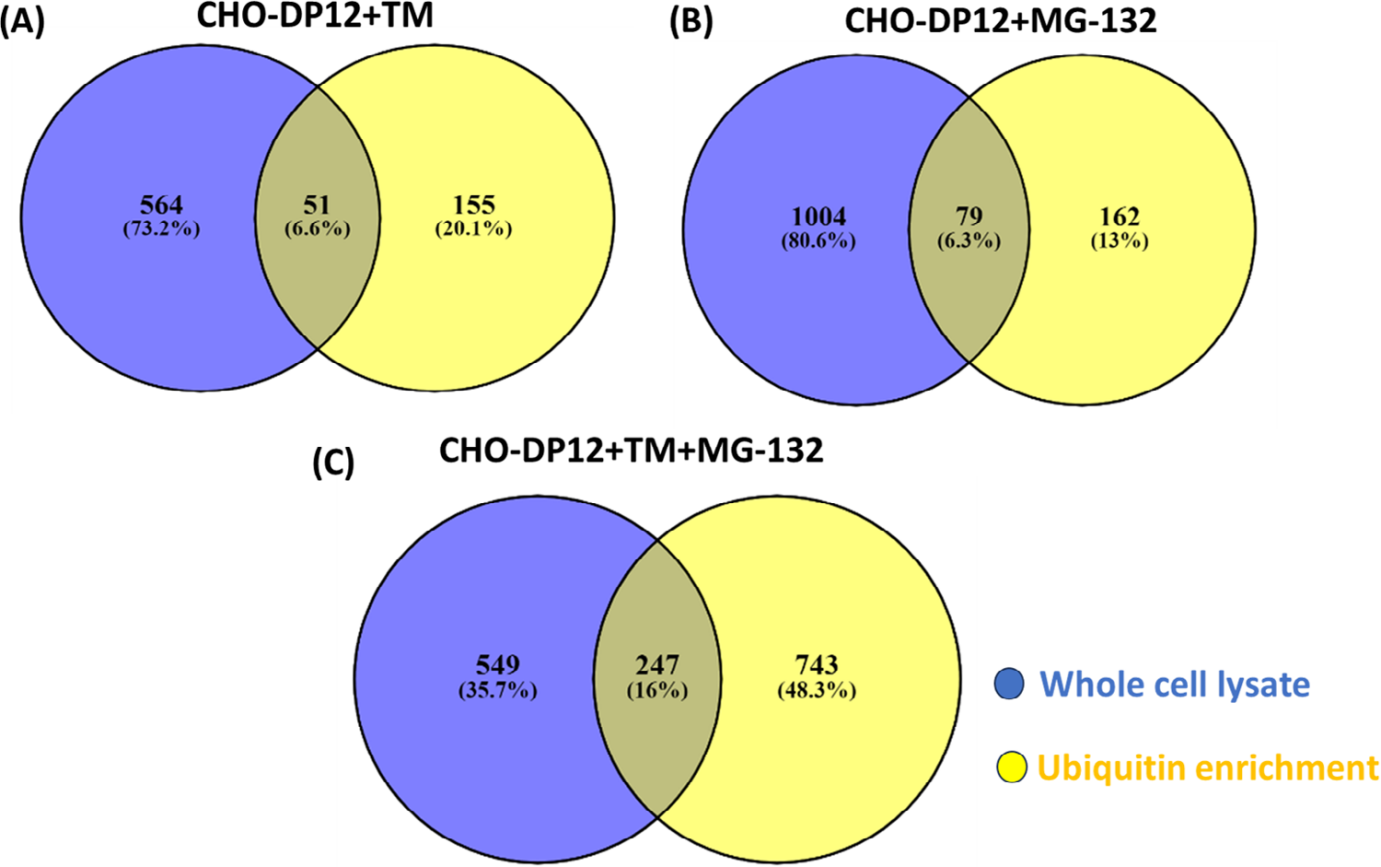
Venn diagram showing the number of shared differentially expressed proteins from CHO‐DP12 whole cell lysate proteomic analysis and the ubiquitinated proteomic analysis following treatment with (A) TM, (B) MG132, and (C) TM + MG132 combined. CHO, Chinese hamster ovary; TM, tunicamycin.

The differential expression analysis of whole cell lysate and K‐ε‐GG‐enriched samples from CHO‐DP12 cells, as well as their TM, MG132, and TM + MG132 treated counterparts, revealed significant changes in protein abundance. Specifically, 239, 375, and 366 proteins (Supporting Information File S2) in the whole cell lysate, and 208, 241, and 979 proteins in the K‐ε‐GG‐enriched samples (Supporting Information File S3), showed altered abundance levels for the DP12 + TM, DP12 + MG132, and DP12 + TM + MG132 experimental groups, respectively (ANOVA *p* < 0.05, fold change ≥1.5, unique peptides ≥ 2 for whole cell lysate, and unique peptides  ≥1 for K‐ε‐GG‐enriched analysis). This is summarized in Table 1. The data reveals significant alterations in protein abundance levels upon ER stress and proteasome inhibition, providing valuable information about the global changes in the ubiquitinome and cellular response to these treatments in CHO cells. We further investigated the overlap between proteins identified through whole cell total proteomic analysis and proteins identified from ubiquitin peptide enrichments from each treatment and included the one peptide assigned to a protein identification from the whole cell lysate proteomic results. The overlap analysis revealed 51 proteins that are shared between the proteomic and ubiquitinated proteomic lists (Figure 3A). The overlapped proteins were further increased to 79 when including the MG132 treatment (Figure 3B) and increased to 247 from the TM + MG132 combination (Figure 3C). In our study, we observed a limited overlap between the nonenriched whole cell lysate proteomic data and the ubiquitinated proteomic data. It should be noted that 155 (TM), 162 (MG132), and 743 (TM + MG132) proteins were uniquely identified in the ubiquitinated differential expression analysis which can be attributed to the crucial necessity for an enrichment

**TABLE 1:** Number of proteins with altered abundance levels in CHO‐DP12 cells after treatment with TM, MG132, and TM + MG132 combined compared to untreated control cells.

Abundance change	CHO‐DP12 + TM	CHO‐DP12 + MG132	CHO‐DP12 + TM + MG132
*Whole cell lysate*			
*Up*	127	154	230
Down	112	221	136
Ubiquitinated proteins			
Up	167	204	884
Down	41	37	95

Abbreviations: CHO, Chinese hamster ovary; TM, tunicamycin.

### 3.4 Pathway Analysis of Ubiquitinated Proteins With Altered Abundance Levels

**FIGURE 4:**
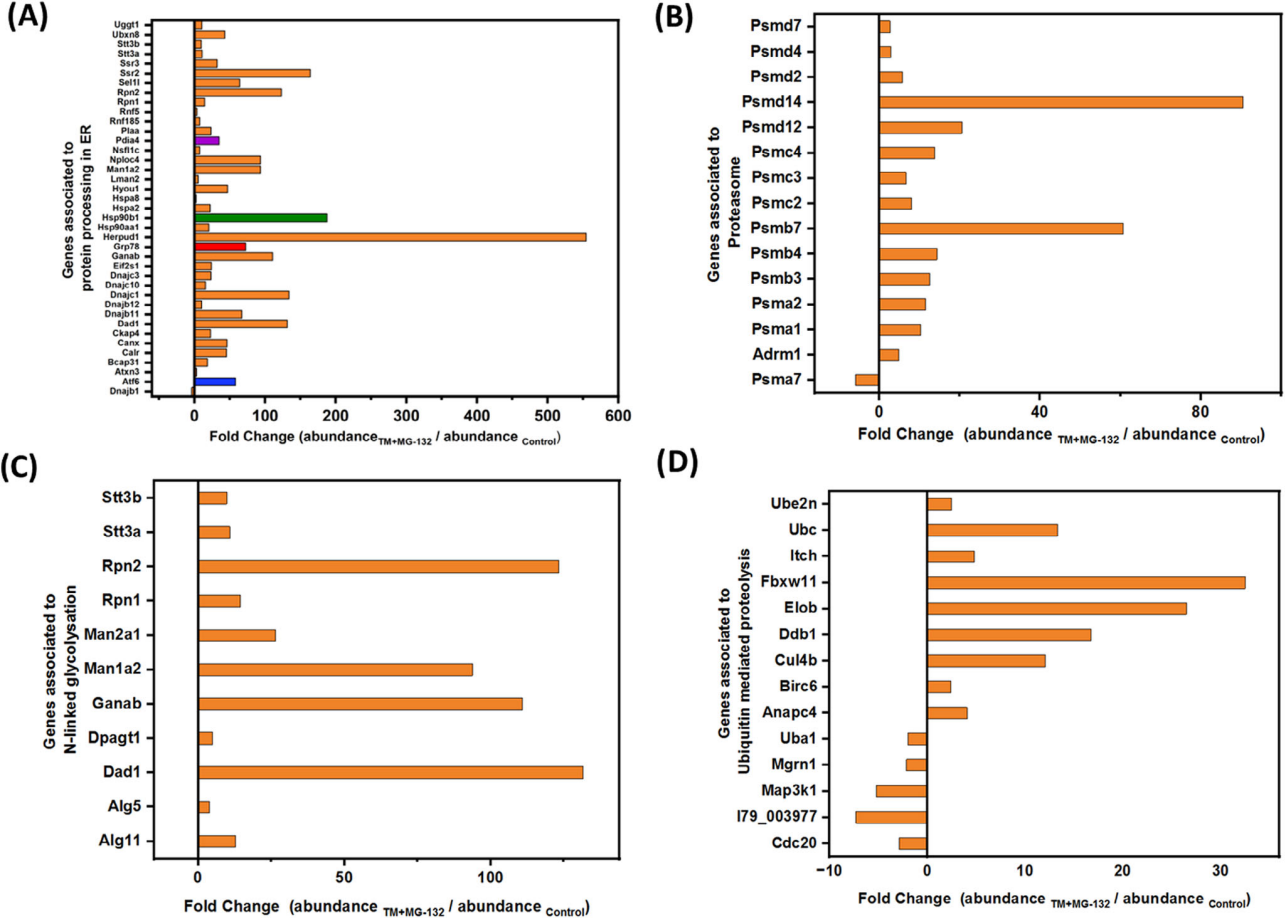
Fold changes of differentially expressed proteins associated to KEGG pathways including (A) protein processing in ER, (B) proteasome, (C) N‐linked glycosylation, and (D) ubiquitin‐mediated proteolysis. x‐axis represents the fold change and y‐axis corresponds to gene symbols associated to KEGG pathways. Highlighted proteins in (A) include GRP78 (red), HSP90B1 (green), ATF6 (blue), and PDIA4 (purple). ER, endoplasmic reticulum; KEGG, Kyoto Encyclopedia of Genes and Genomes.

To explore the pathways that are involved in the stress response of CHO‐DP12, GO analysis was performed. As the ubiquitinated proteome of CHO cell lines remains largely unexplored, we therefore specifically focused on pathways involving ubiquitinated proteins with altered abundance following induction of ER stress. The STRING GO analysis of ubiquitinated proteins with altered abundance levels from CHO‐DP12 following exposure to TM + MG132 showed a significant enrichment of ubiquitinated proteins involved in KEGG pathways such as “proteasome” (cge03050), “protein processing in endoplasmic reticulum” (cge04141), “*N*‐glycan biosynthesis” (cge00510), and “ubiquitin mediated proteolysis” (cge04120) (Table 2). Further pathway analysis revealed a significant enrichment of ubiquitin‐related “biological processes” such as “protein ubiquitination”, “proteasome‐mediated ubiquitin‐dependent protein catabolic process”, and “ubiquitin‐dependent ERAD pathway” (Table 3). Figure 4 shows the fold changes in abundance levels of these ubiquitinated proteins associated with the above pathways.

**TABLE 2:** KEGG pathway enrichment analysis of differentially expressed ubiquitinated proteins associated to K‐ε‐GG peptides in CHO‐DP12 cells following treatment with TM + MG132 combined.

Pathway ID	Enriched KEGG pathways	Count in network/total	Identified DE proteins	Strength	FDR
cge03050	Proteasome	15/37	Psma7, Psmc3, Psmd4, Psmb4, Psmb3, Psmd12, Psmc2, Psma2, Psmb7, Psmd14, Psma1, Psmd2, Psmd7, Psmc4, Adrm1	1	3.64 × 10^−8^
cge04141	Protein processing in ER	39/128	Eif2s1, Uggt1, Sel1l, Calr, Herpud1, Canx, Hsp90b1, Hyou1, HSPA5, Nploc4, Dnajc10, Stt3b, Rpn2, Ssr3, Hsp90aa1, Dnajb11, Stt3a, Atf6, Ganab, Rpn1, Pdia4, Dnajc3, Hspa2, Man1a2, Hspa8, Bcap31, Ssr2, Dnajb12, Dnajc1, Dnajb1, Nsfl1c, Atxn3, Ubxn8, Plaa, Rnf5, Rnf185, Dad1, Lman2, Ckap4	0.87	4.07 × 10^−17^
cge00510	*N*‐Glycan biosynthesis	11/45	Stt3b, Rpn2, Alg5, Rpn1, Stt3a, Dpagt1, Ganab, Dad1, Alg11, Man2a1, Man1a2	0.78	0.00011
cge04120	Ubiquitin mediated proteolysis	14/104	I79_003977, Birc6, Cdc20, Ube2n, Ubc, Itch, Ddb1, Anapc4, Map3k1, Uba1, Cul4b, Elob, Mgrn1	0.52	0.0016

*Note*: Strength: Log10(observed/expected). This measure describes how large the enrichment effect is. It is the ratio between (i) the number of proteins in the network that are annotated with a term and (ii) the number of proteins that are expected to be annotated with this term in a random network of the same size.

Abbreviations: CHO, Chinese hamster ovary; ER, endoplasmic reticulum; KEGG, Kyoto Encyclopedia of Genes and Genomes; TM, tunicamycin.

**TABLE 3:** GO biological process enrichment analysis of differentially expressed ubiquitinated proteins associated to K‐ε‐GG peptides in CHO‐DP12 following treatment with TM + MG132 combined.

GO ID	Biological process	Count in network/total	Strength	FDR
GO:0097039	Protein linear polyubiquitination	3/3	1.39	0.0216
GO:0090611	Ubiquitin‐independent protein catabolic process via the multivesicular body sorting pathway	3/5	1.17	0.0470
GO:1903069	Regulation of ER‐associated ubiquitin‐dependent protein catabolic process	4/8	1.09	0.0184
GO:0043162	Ubiquitin‐dependent protein catabolic process via the multivesicular body sorting pathway	9/29	0.88	0.00057
GO:0030433	Ubiquitin‐dependent ERAD pathway	19/81	0.76	9.4 × 10^−7^
GO:0070534	Protein K63‐linked ubiquitination	8/29	0.7	0.0377
GO:0043161	Proteasome‐mediated ubiquitin‐dependent protein catabolic process	59/334	0.63	1.94 × 10^−16^
GO:0016574	Histone ubiquitination	10/42	0.76	0.0011
GO:0006511	Ubiquitin‐dependent protein catabolic process	90/552	0.6	2.4 × 10^−23^
GO:0032434	Regulation of proteasomal ubiquitin‐dependent protein catabolic process	19/132	0.55	0.00038
GO:0032436	Positive regulation of proteasomal ubiquitin‐dependent protein catabolic process	12/89	0.52	0.0135
GO:0031397	Negative regulation of protein ubiquitination	14/99	0.54	0.0041
GO:2000058	Regulation of ubiquitin‐dependent protein catabolic process	22/175	0.49	0.00052
GO:2000060	Positive regulation of ubiquitin‐dependent protein catabolic process	13/103	0.49	0.0141
GO:0016579	Protein deubiquitination	18/141	0.49	0.0020
GO:0031396	Regulation of protein ubiquitination	22/232	0.36	0.0111
GO:0016567	Protein ubiquitination	58/650	0.34	9.3 × 10^−06^

*Note*: The table lists ubiquitin‐related biological processes that are enriched from the differentially expressed proteins.

Abbreviations: CHO, Chinese hamster ovary; ER, endoplasmic reticulum; ERAD, endoplasmic reticulum‐associated degradation; GO, Gene Ontology; TM, tunicamycin.

For a comprehensive proteomic investigation into the mechanisms associated with ER stress in CHO‐DP12 cells, further exploration of the “Protein processing in ER” pathway was conducted. The network of ubiquitinated proteins with changes in abundance levels associated with “Protein processing in ER” and their corresponding fold changes showed that the most substantial alterations are observed in the abundance levels of GRP78, HSP90B1, HERPUD1, ATF6, GANAB, and PDIA4. Figure 4A shows altered abundance levels of a number of the proteins associated with “Protein processing in the ER” including GRP78, ATF6, HSP90B1, and PDIA4. It should be noted that these stress response proteins identified in the “Protein Processing in ER” pathway exhibited significant increases in abundance levels of the ubiquitinated form of these proteins (see Figure S1). For example, GRP78 plays a central role in ER homeostasis and the UPR by providing translocation of nascent proteins across the ER membrane, directing protein folding, and directing misfolded proteins to ERAD and the proteasome [48]. In our study, GRP78 was detected to be 36.6‐fold higher in TM‐treated cells compared to untreated controls, whereas this difference increased to 72.6‐fold between TM + MG132 treated cells and untreated cells (Supporting Information File S3). In nonstress conditions, GRP78 binds and stabilizes the ER‐transmembrane signaling molecules (ATF6, IRE1, and PERK), whereas it is cleaved and titrated away from these molecules under ER stress. The increased amount of ubiquitinated GRP78 protein levels can be attributed to the activation of ER stress in TM‐treated cells. For the cells that were treated with TM + MG132, the higher increase comes from the cumulative effect arising from cleaved and ubiquitinated GRP78 and the inability of the cells to remove ubiquitinated GRP78 due to proteasomal inhibition. It is important to note that the differential whole cell lysate proteomic data obtained from the comparison of TM and TM + MG132‐treated cells to untreated controls showed an equal amount of change in the abundance of GRP78 (Supporting Information File S2). In both conditions, GRP78 was detected to be ∼4‐fold higher in treated cells compared to nontreated ones, showing the importance of the investigation of PTMs to delve deeper into the role of target proteins. In cells exposed solely to MG132, no significant difference was observed in the abundance of GRP78. This finding remained consistent for both whole cell lysate (Supporting Information File S2) and ubiquitin‐enriched samples (Supporting Information File S3), with a 1.6‐fold increase noted in MG132‐treated cells.

ATF6 plays a crucial role in the survival mechanisms of cells by regulation of expression levels of ER chaperones GRP78 and GRP94 as well as the expression of PDIs. Differential analysis of the ubiquitinated proteome showed a significant increase in the abundance of ubiquitin‐modified ATF6 in TM and TM + MG132 treated cells compared to untreated control. Pairwise differential analysis of TM and control cells revealed that ubiquitinated ATF6 was present upon TM treatment, whereas it was not detected in control cells. When cells were treated with a combination of TM and MG132, ubiquitinated ATF6 protein levels were detected to be 58‐fold higher in treated cells compared to untreated controls (Figure S1). ATF6 was not detected in the whole cell lysate without ubiquitination enrichment, once again demonstrating the importance of utilizing PTM information to understand mechanisms involved in cellular stress. Although the mechanism of ubiquitin‐mediated acute degradation of ATF6 is limited, ATF6 has been shown to be a direct target of the UPS during ER stress [49]. A recent study found that the ER‐localized E3 ligase, RNF186 catalyzes the nonproteolytic ubiquitination of K152 on ATF6, thereby improving the UPR and underscoring the key role of ATF6 ubiquitination [50]. Our study also shows that acute induction of ER stress via TM activates the ubiquitination of ATF6 in CHO‐DP12 cells, and this effect multiplies with the inhibition of proteasome degradation.

The protein disulfide isomerase (PDI) family of proteins located within the ER plays a crucial role in the folding and assembly of proteins within the ER, catalyzing the formation, cleavage, and subsequent reformation of disulfide bonds [51]. Moreover, PDI is also known for its chaperone‐like activity [52]. PDI consists of catalytic domains (active sites), each of which incorporates the canonical CGHC motif, along with noncatalytic domains [53]. Due to the substantial number of disulfide bonds present in antibodies, PDIs play an important role in facilitating their assembly [54]. It has been reported that under ER stress, a significant increase in the levels of PDIA3 and PDIA4 proteins has been observed and was found to affect the production of mAbs in CHO cells [55, 56]. During ER stress, PDIA3 cleaves unfolded antibody heavy chain (HC) molecules, expediting the dislocation of the ubiquitinated N‐terminal domain of HC for ERAD‐mediated degradation [57]. This process leaves the remaining C‐terminal domain for further ubiquitination by ubiquitin protein ligases such as UBR4/UBR5. Our study has shown the abundance of nonubiquitinated PDIA3 in the whole cell lysate remains similar in TM (1.6‐fold), MG132 (1.7‐fold), and TM + MG132 (1.6‐fold)‐treated CHO‐DP12 cells (Supporting Information File S2), whereas an increase in the abundance of ubiquitinated PDIA3 in TM (12‐fold), MG132 (110‐fold), and TM + MG132 (58‐fold)‐treated CHO‐DP12 cells compared to untreated controls (Supporting Information File S3). Our study revealed a notable increase in the abundance of ubiquitinated PDIA3 levels when proteasomal activity was inhibited. In addition, PDIA4 was found to be 35‐fold higher in TM + MG132 treated cells compared to the untreated control cells. Along with PDIs, significant high fold changes were observed for ubiquitin protein E3 ligases such as UBR2 (3.8‐fold) and UBR4 (39‐fold). Further investigation on the role of ubiquitinated PDIAs associated with ubiquitin protein E3 ligases will be necessary to understand its relationship to the productivity of mAbs by CHO cells.

## 4 Conclusion

The identification of many ubiquitinated proteins with altered levels of abundance following induction of ER stress emphasizes the significance of incorporating ubiquitination studies in the proteomic analyses of CHO cell factories. These studies have been largely overlooked in CHO ‘omics studies until now. The incorporation of ubiquitinated proteomic data alongside whole cell proteomic data has significantly enhanced overall proteome coverage. For example, ATF6 was identified as showing altered levels of abundance of the ubiquitinated ATF6 protein but was not detected in the whole cell lysate proteomic data (see Figure S1). In this study, we observed that the ER stress response proteins, including GRP78, HSP90B1, ATF6, PDIA3, and PDIA4, demonstrated a pronounced level of ubiquitination following induction of ER stress. Furthermore, these proteins exhibited a substantial upregulation in abundance levels, signifying a heightened cellular response to ER stress. To date, this is the first in‐depth analysis of the ubiquitinated proteome of CHO cells under ER stress conditions. The findings from this study could contribute significantly to our understanding of cellular responses to ER stress, which are crucial for optimizing bioprocess conditions, thereby enhancing protein productivity. For example, the study has shown how CHO cells respond to ER stress, highlighting the role of enriched KEGG pathways such as “Protein processing in the ER” in this process. This knowledge would allow for better manipulation of pathways such as the UPR and ERAD to reduce misfolded proteins, improve folding efficiency, and optimize cellular health during bioproduction processes, ultimately leading to higher yields and more consistent protein quality in industrial applications. Future work aims to quantify protein expression and ubiquitination changes through targeted mass spectrometry, specifically employing Parallel Reaction Monitoring for detailed analysis [56].

## Author Contributions

**Karuppuchamy Selvaprakash**: investigation (supporting), methodology (supporting), writing—original draft (supporting). **Christiana‐Kondylo Sideri**: investigation (supporting), methodology (supporting), writing—review and editing (supporting). **Michael Henry**: data curation (supporting), methodology (supporting), software (supporting), supervision (supporting), writing—review and editing (supporting). **Esen Efeoglu**: investigation (supporting), methodology (supporting), supervision (supporting), writing—review and editing (supporting). **David Ryan**: investigation (supporting), methodology (supporting), writing—review and editing (supporting). **Paula Meleady**: conceptualization (lead), funding acquisition (lead), project administration (lead), writing—review and editing (lead).

## Conflicts of Interest

The authors declare no conflicts of interest.

## Data Availability

The authors have nothing to report.
